# The unrecognized occupational relevance of the interaction between engineered nanomaterials and the gastro-intestinal tract: a consensus paper from a multidisciplinary working group

**DOI:** 10.1186/s12989-017-0226-0

**Published:** 2017-11-25

**Authors:** Antonio Pietroiusti, Enrico Bergamaschi, Marcello Campagna, Luisa Campagnolo, Giuseppe De Palma, Sergio Iavicoli, Veruscka Leso, Andrea Magrini, Michele Miragoli, Paola Pedata, Leonardo Palombi, Ivo Iavicoli

**Affiliations:** 1 Department of Biomedicine and Prevention, University of Rome Tor Vergata, Via Montpellier 1, 00133 Rome, Italy; 2 Department of Sciences and Public Health and Pediatrics, University of Turin, Turin, Italy; 3 Department of Medical Sciences and Public Health, University of Cagliari, Cagliari, Italy; 4 Department of Medical and Surgical Specialties, Radiological Sciences, and Public Health, Section of Public Health and Human Sciences, University of Brescia, Brescia, Italy; 5 Department of Occupational and Environmental Medicine, Epidemiology and Hygiene, Italian Workers’ Compensation Authority (INAIL), Rome, Italy; 6 Department of Public Health, University of Naples Federico II, Naples, Italy; 7 Department of Medicine and Surgery, University of Parma, Parma, Italy; 8 Department of Experimental Medicine- Section of Hygiene, Occupational Medicine and Forensic Medicine, University of Campania Luigi Vanvitelli, Naples, Italy

**Keywords:** Ingested nanoparticles, Inhaled nanoparticles, Direct toxicity, Indirect toxicity, Workers’ exposure, Gastrointestinal tract, Microbiota

## Abstract

**Background:**

There is a fundamental gap of knowledge on the health effects caused by the interaction of engineered nanomaterials (ENM) with the gastro-intestinal tract (GIT). This is partly due to the incomplete knowledge of the complex physical and chemical transformations that ENM undergo in the GIT, and partly to the widespread belief that GIT health effects of ENM are much less relevant than pulmonary effects.

However, recent experimental findings, considering the role of new players in gut physiology (e.g. the microbiota), shed light on several outcomes of the interaction ENM/GIT. Along with this new information, there is growing direct and indirect evidence that not only ingested ENM, but also inhaled ENM may impact on the GIT. This fact, which may have relevant implications in occupational setting, has never been taken into consideration.

This review paper summarizes the opinions and findings of a multidisciplinary team of experts, focusing on two main aspects of the issue: 1) ENM interactions within the GIT and their possible consequences, and 2) relevance of gastro-intestinal effects of inhaled ENMs. Under point 1, we analyzed how luminal gut-constituents, including mucus, may influence the adherence of ENM to cell surfaces in a size-dependent manner, and how intestinal permeability may be affected by different physico-chemical characteristics of ENM. Cytotoxic, oxidative, genotoxic and inflammatory effects on different GIT cells, as well as effects on microbiota, are also discussed.

Concerning point 2, recent studies highlight the relevance of gastro-intestinal handling of inhaled ENM, showing significant excretion with feces of inhaled ENM and supporting the hypothesis that GIT should be considered an important target of extrapulmonary effects of inhaled ENM.

**Conclusions:**

In spite of recent insights on the relevance of the GIT as a target for toxic effects of nanoparticles, there is still a major gap in knowledge regarding the impact of the direct versus indirect oral exposure. This fact probably applies also to larger particles and dictates careful consideration in workers, who carry the highest risk of exposure to particulate matter.

## Background

Despite the large and growing number of ENM used in agri-food products [1–5], oral ingestion has received significantly less attention than the pulmonary route and therefore there is relatively lower information on the possible toxic effects of ENM on the gastro-intestinal tract (GIT). This may be due to the fact that the study of the impact of ENM on the GIT (and vice versa) is a rather complicated issue: both food and the processes that break down and transform food ingredients (e.g., physical forces, osmotic concentration and pH gradients, digestive enzyme, redox conditions and salinity levels) may in fact transform, aggregate and dissolve ENMs in ways that alter their naive and inherent properties, therefore potentially affecting their biological reactivity as well as their toxicological profiles.

This picture is however changing: It is becoming clear that the gut micro-organisms (the microbiota) play a pivotal role in maintaining both local (intestinal) and systemic homeostasis and that they may influence ENM and be influenced by them [6, 7]. Very recent in vitro and in vivo data, discussed in the first section of the present review, have shown that ingested ENM may induce substantial adverse effects unrecognized in past studies; last but not least, there is indirect growing evidence that inhaled ENM, representing the most common pathway of exposure in workers, may have a substantial impact on the GIT, as shown in the second section of the review.

In September 2016, the Italian Society of Occupational Medicine and Industrial Hygiene (SIMLII) hosted a research workshop in order to exchange and merge knowledge and expert point of view on the above-mentioned topics. In the following sections, we outline how these topics have been developed and summarize the state of the evidence about their possible impact on future research in the field of nanotoxicology.

## Interaction of ingested ENM with the GIT

### Aggregation, agglomeration and dissolution

The fate and bioavailability of ENM in the gastrointestinal system may be affected, at least partly, by their primary characteristics, such as size, surface chemistry and charge, or, in turn, by properties acquired through the transit via the GIT. Several factors, such as pH gradients, gastrointestinal transit time, nutritional status, meal quality, level of mucosal and enzymatic secretions, as well as the intestinal microflora, may all influence ENM physical and chemical reactivity [8]. There is limited information on the physical changes of some metallic ENM (Ag, TiO2, SiO2 and ZnO) once in contact with the gastro-intestinal fluids. It seems that ion release may occur in the gastric environment, along with size-dependent aggregation and agglomeration. For example, it has been shown that in gastric juice ZnO and Ag undergo dissolution [9–14]. Agglomeration has been shown for TiO2 and also for Ag ENM, dependently from size [9–13, 15, 16]. Conflicting data have been obtained as far as aggregation/agglomeration in the intestinal environment is concerned: agglomeration has been reported for SiO_2_ [17] and de-agglomeration for Ag [9]. Probably the chemical composition of ENM, their surface charge and the fasting/fed state may be important components of the final outcome. Clearly, more data are needed in order to understand how different variables such as previous transit in different environments, fasting and fed state may each contribute to the final physical status of ENM travelling along the GIT. In addition, a higher number of ENM and of gastro-intestinal physiological states should be investigated.

### ENM uptake and absorption

Although limited information is available on the toxicokinetics of orally administered ENM [18], available data suggest that uptake and absorption of ENM in the GI tract may have relevant implications for their local and systemic effects [19, 20].

A detailed description of the GIT cellular and extracellular structures involved in the uptake and absorption of ENMs, and of the mechanisms of uptake are beyond the scope of this review, however a brief presentation of the main players is needed in order to understand the fate of ENM in the intestine.

In this regard, the key cell types are a) the enterocytes, which are by far the most represented cell type along the intestine and are connected each other by tight junctions, which prevent the unselected intercellular access to the luminal content; b) the antigen sampling M cells, overlying organized lymphoid structures such as the Peyer’ s patches and other gut-associated lymphoid tissue (GALT). Although representing only 1% of the intestinal cells, M cell are covered by a much thinner mucus layer than enterocytes, and are very relevant for the uptake of foreign substances, which are subsequently delivered to the underlying lymphoid cells; c) the mucus producing goblet cells (about 10% of the total intestinal cells), secreting the mucus lining the whole surface of the small and large intestine.

The first barrier encountered by ingested ENM is indeed represented by mucus, which has been reported to be efficient in trapping larger ENM [21], this factor being a possible explanation for the less pronounced toxic effects of 200 nm Ag ENM in comparison to 20 nm Ag ENM observed in in vitro experiments on a co-culture of CaCo 2 cells and mucus producing cells [22]. Ingested ENM may on the other hand influence mucus secretion, in both quantitative and qualitative terms. For example, sub-chronic (28-days) oral exposure to 60 nm Ag ENM in rats [23] promoted the secretion of mucus in the ileum and rectum, and changes in mucin composition (amounts of neutral and acidic mucins and proportions of sulfated and sialylated mucins). This may be interpreted as a non-specific inflammatory response.

Once crossed the mucus barrier, ENM come in contact with the intestinal cells: the main mechanism through which they may cross the intestinal barrier is represented by transcellular transport. Available in vitro studies suggest that smaller particles may traverse enterocyte cell membranes, mediating changes in membrane fluidity, resulting in altered signaling or increased permeability and cytotoxicity; conversely, as particle size increases or as agglomeration occurs, uptake is predominantly performed by M cells, which are already specialized for this function [20]. Of note, the immunologic responses by lymphoid tissue beneath M cells is typically oriented to hypo-responsiveness (oral tolerance). It is not known, however, whether environmental ENM can have similar mucosal immunologic effects. Evidence for this possibility arises from the observation that agglomerates of endogenous calcium-phosphate nanoparticles (of similar size to ENM in biological media) and dietary TiO_2_ can bind gut microbial-derived molecules (e.g. peptidoglycan, lipopolysaccharide) and traffic these to GALT, with influence on tolerance or immunogenicity [24–27].

In vivo studies in rodents suggest that a low percentage of ENM present in the gut lumen is actually absorbed. For example, in a long term study (24 or 84 days) of orally administered amorphous silica (7 nm or 10–25 nm), absorption was 0.25% [28]. A higher uptake was reported in another 10 day administration study, in which 500 nm TiO_2_ particles, given by gavage, were taken up in percentages ranging from 0.11%, in the stomach, to 4% in the large intestine, and the vast majority of the ENM accumulated in the Peyer’s patches [29]. Although the size of TiO_2_ particles in the above-mentioned study was beyond the conventional size limit of ENM (100 nm), the findings of another report, showing the presence of 12 nm TiO_2_ particles in Peyer’s patches soon after a single administration by gavage, suggest that early absorption of ENM occurs [15]. Of relevance, available information from in vitro experiments suggests that uptake of ENM may be decreased by food components, as shown for silica and polystyrene [17]. Some data regarding TiO_2_ upake and absorption, after a single administration, are available also for humans, and they range from no evidence of absorption (TiO_2_ size: 10–1800 nm; dose range 315–620 mg) [30] to detectable elemental Ti in blood after the administration of 100 mg of 260 nm particles [31].

Data regarding chronic low dose exposure are of course needed in order to clarify the presence and extent of intestinal absorption in humans under real life exposure settings.

### Toxic effects

In the following paragraphs the most relevant available in vitro and in vivo studies on ENM toxicity on the GIT are summarized.

#### In vitro studies

##### Cell damage

In Caco-2 cells, TiO_2_ ENM exposure caused loss and morphological changes in microvilli and disorganization of the brush border [32], while rutile-cored aluminum hydroxide and polydimethylsiloxane-surface treated TiO_2_ ENM did not cause any damage [33]. Epithelial alterations, consisting of plasma membrane disruption and tight junction loosening, have been demonstrated also by Mahler et al. [34] in a tri-culture gut model including enterocytes, goblet cells and M cells, treated with 50 and 200 nm polystyrene beads.

##### Changes in permeability

Changes in permeability of the epithelial barrier may be interpreted as the result of functional damage to the integrity of the intestinal barrier, sometimes preceding the development of evident cellular damage. Ag ENM treatment of T84 human colonic epithelial cells, characterized by polarized monolayers naturally producing mucus, induces size- and dose-dependent changes in the expression of genes involved in anchoring tight junctions, which results in increased intestinal permeability [35]. A significant increase in epithelial permeability of Caco-2 tight monolayers was reported also for TiO_2_ ENM [36] and a reversible effect was also observed for differently functionalized fullerenes and single walled carbon nanotubes (SWCNTs) [37].

However, other studies performed on Caco-2 monolayers as well as on Caco-2/HT29-MTX co-culture models failed to detect such alterations both for TiO_2_ [15], SiO_2_ [17] and Ag ENM [38].

These conflicting findings may be explained by the different doses, in vitro models, methods of detection and physico-chemical characteristics of the tested ENM. As in other experimental settings regarding the study of toxicity of ENM, grouping of ENM and standardized experimental conditions may help to clarify the role of different ENM in inducing alterations of intestinal permeability.

##### Cell viability and proliferation

Different culture methods have been used in order to study the effects of ENM on viability of cells of the GIT. Since different models may show different sensitivity, we are presenting separately data regarding undifferentiated and differentiated mono-cultures and those regarding co-culture models.

##### Studies in undifferentiated mono-cultures

These studies generally show alterations in cell viability induced by metal based ENM, with high cytotoxicity induced by ZnO [39–43], SiO_2_ [17, 44, 45], and Ag ENM [46]. Milder cytotoxic effects have been reported for Au [47–49], TiO_2_ ENM [15, 32, 33, 40, 48, 50–56], and carbon nanotubes [37, 51, 57, 58] in short-term studies.

##### Studies in fully differentiated Caco-2 cells cultures

Relatively few studies are available in fully differentiated Caco-2 cells, which, however, better reflect the native GIT and are generally less sensitive to cytotoxic injuries [45, 59]. Nevertheless, some ENM such as Ag and ZnO ENM [45] are equally toxic to both undifferentiated and differentiated cultures. It remains to be defined whether the effect is attributable to ENM themselves, or to ion release or to both [60].

##### Studies in co-culture models

Toxicity of nanomaterials (TiO_2_ NM101, Ag NM300, Au) has been evaluated in non-inflamed and inflamed co-cultures, and also compared to non-inflamed Caco-2 monocultures. The inflamed co-cultures released higher amounts of IL-8 compared to Caco-2 monocultures, but the cytotoxicity of Ag NP was higher in Caco-2 monocultures than in 3D co-cultures [48]. However, other investigations failed to detect such differential vulnerability of Caco-2 monocultures to Ag ENM [22, 39, 61–64]. Nevertheless Ag ENM were found to be more toxic than TiO2 or Au ENM [48], while negligible toxic effects have been reported for Carbon nanotubes [65, 66]. More complex in vitro intestinal models have been proposed, such as organoid cultures; these seem very promising for studies on diseased gut, however such models are not completely characterized yet [20].

When investigating in vitro the potential toxicity of ENM on the GIT, several in vivo occurring phenomena should be considered and reproduced to more faithfully mimic the in vivo conditions. As already discussed, there is an ongoing debate on the contribution of the time-dependent dissolution and ion release from metal based ENM to cytotoxic effects [60].

Moreover, the modifications induced to ENM after the interactions with different GIT compartments should be carefully addressed. For instance, acid treatment simulating quantum dots (QD) exposure to gastric juice increased the toxicity of PEG-coated QDs on Caco-2 cells, as a consequence of coating removal, which enabled dissolution into Cd^2+^ ions [67]. Conversely, simulation of Ag ENM digestion, with or without organic and food components, did not significantly affect cytotoxicity and only caused minor agglomeration of particles [13, 38]. Therefore, considering that pH in vivo varies across different gut compartments and with composition of ingesta, future research should aim to clarify whether and to what extent these conditions may affect ENM toxicity. The effect of food should also be considered. A paradigmatic example is that of micronutrients; whereas phenolic compounds (namely, quercetin and kaempferol), present in fruits and vegetables, can protect Caco-2 cells from Ag ENM induced toxicity and thus maintained the integrity of the epithelial barrier, resveratrol do not exert such effects [68, 69]. This protective action may be attributed to the potent anti-oxidant properties of flavonoids. Another study failed to detect differences in cytotoxicity between digested or undigested Ag ENM on Caco-2 cells when the digestion process was implemented with the presence of the main food components, i.e. carbohydrates, proteins and fatty acids [38, 59]. Native TiO_2_ ENM and TiO_2_ ENM pretreated with digestion simulation fluid or bovine serum albumin did not show significant different toxicity in Caco-2 cells [59]**.** The administration of ZnO NPs in combination with fatty acids, on the other hand, increased their cytotoxic effects [70]. Overall, these data strongly support the relevance, when investigating the potential toxicity of orally ingested ENM, of developing in vitro models which take into account the possible ENM transformation after contact with food or food components, with acidic pH, and GIT constituents in order to mimic in vivo realistic scenarios.

Studies regarding effects of ENM on cell viability are detailed in Table 1, where doses, and physico-chemical characteristics of tested ENM are also reported. For a more complete overview of the available literature, the table includes also studies that have not been discussed in the text.

**Table 1:** Studies addressing the effects of different NPs on viability of gastro-intestinal cellular models

Nanoparticles	Physico-chemical NP properties	Cell line	Experimental design	Cytotoxic effects	Reference
SW-CNTs	Surface functionalization: SW-CNTs modified with COOH-functional groups.	SW480	Up to 24 h exposure to CNTs (0.5–2 μg per well)	After 4 and 24 h of exposure, CNTs did not have any cytotoxic effect, however there was a reduction in viability at 48 h and for the highest dose employed.	Kulamarva et al. 2008 [58]
CdSe- QDs; CdSe-ZnS-PEG coated QDs	Surface characterization: ZnS shell and poly-ethylene glycol hydrophilic coating.	Caco-2	Twenty four h exposure to native QDs or QDs incubated acidic medium (0–105 nmol/ml)	A dose dependent cytotoxicity for CdSe-QDs was detected. Toxic effects increased with increasing the Cd/Se ratio during synthesis. PEG-coated QDs had less effects. The relative viability of Caco-2 cells dropped from 90% when incubating with 4.2 nmol/ml CdSe-ZnS-PEG-QDs to 53% when incubating with acid medium treated QDs. This result was not confirmed for CdSe- QDs incubated into acid medium.	Wang et al. 2008 [67]
Au-nanorods	CTAB capped and PAA and PAH-coated Au-nanorods.	HT-29	Four days exposure to Au-nanorods (0.4 nM)	CTAB-capped Au-nanorods displayed a significant cytotoxicity (65–75% loss of viability), independent of the aspect ratio. PAA- and PAH-coated gold nanorod solutions were found relatively nontoxic	Alkilany et al. 2009 [49]
SW- CNTs	Size: average diameter of individual SW-CNT is 1.4 ± 0.1 nm, bundle dimensions are 4–5 nm × 0.5–1.5 μm Surface functionalization: carboxylic acid.	Caco-2	Twenty-four h exposure to CNTs (5 and 1000 μg/ml)	A significant decrease in cell viability was detected at the higher concentrations: 500 and 1000 μg/ml.	Jos et al. 2009 [57]
MW-CNTs	Impurities: traces of cobalt, nickel, zinc and lead	Caco-2	Seventy-two h exposure to MW-CNTs (0–100 μg/ml)	No significant difference in CFE dose-effect relationship in comparison to controls.	Ponti et al. 2010 [65]
ZnO-NPs	Size: 50–70 nm; Average diameter: 196 nm; Surface area: 3 m^2^/g.	Lovo	Up to 72 h exposure to ZnO, (0–23 μg/ml)	ZnO-NPs induced a time- and dose-dependent decrease of cell number. Ten, 20 and 40 μg/cm^2^ induced <5% cell survival after 24 h. Dose-dependent apoptotic cell death was evident.	De Berardis et al. 2010 [42]
TiO_2_-NPs	Size: <40 nm; Crystal form: a mixture of rutile and anatase; Surface area: 20–40 m^2^/g; Hydrodynamic diameter (water): 220 ± 20 nm.	Caco-2	Twenty-four h, or 10 days chronic exposure to TiO_2_-NPs (0–1000 μg/ml)	Little indication of any cell fatality compared to the controls was reported at both time points for all concentrations employed.	Koeneman et al. 2010 [32]
Ag-NPs	–	Caco-2	Twenty-four h exposure to Ag-NPs (0–10 μg/ml)	At 1 μg/ml cells did not show a significant viability decrease (LD_50_: ~5 μg Ag/ml).	Lamb et al. 2010 [62]
Ag-NPs	Size (mean ± SD): 20 ± 2–113 ± 8 nm; Hydrodinamic diameter (mean ± SD): - MQ water (24 h followed by sonication): 94 ± 4–177 ± 8 nm; - DMEM (24 h followed by sonication): 118 ± 8–189 ± 9; Impurities in NP suspensions: none. Elementary Ag + in supernatant: 17.4; 15.8; 5.8; and 7.6% for 20, 34, 61, 113 nm NPs, respectively	Caco-2 and Raji B cells in co-culture	Twenty-four h exposure to Ag-NPs (0–50 μg/ml)	No significant viability alterations were observed.	Bouwmeester et al. 2011 [61]
Fe_2_O_3,_ TiO_2_, SiO_2_, and ZnO nano-powders	Size: 3, 5, 10 and 8–10 for Fe_2_O_3,_ TiO_2_, SiO_2_, and ZnO- NPs, respectively. Agglomerate mode (DMEM culture media): 1300, 1000, 600, 650 nm, respectively. Surface area: 222, 240, 124, 24 m^2^/g, respectively.	Caco-2 and RKO	Twenty-four h exposure to SiO_2_, TiO_2_, ZnO and Fe_2_O_3_ nano- powders (0–100 μg/cm^2^) in the presence or absence of TNF-α.	TiO_2_, SiO_2_, and Fe_2_O_3_ had minimal toxicity below 100 μg/cm^2^. ZnO displayed the most relevant toxicity (LC_50_ 27 ± 3.6 and 28 ± 4.6 μg/cm^2^ for RKO and Caco-2, respectively). TNF-α pretreatment did not induce differences in cell viability.	Moos et al. 2011 [41]
Fullerenes; SW-CNTs	Surface functionalization: polyhydroxy small-gap fullerenes (OH-fullerenes), COOH-SW-CNTs; PEG-SW-CNTs. Size: 0.7 (fullerenes); 1.4 ± 0.1 nm in diameter, 4–5 nm × 0.5–1.5 μm bundle dimension (COOH-SW-CNTs); 1.4 ± 0.1 nm in diameter, 4–5 nm × 0.5–0.6 μm in bundle dimension (PEG-SW-CNTs). Purity: >90% (COOH-SW-CNTs); >80% (PEG-SW-CNTs).	Caco-2 cells	Twenty-four h exposure to carbon nanomaterials (0–1000 μg/ml)	All three carbon nanomaterials had minimum cytotoxicity on Caco-2 cells (range of 15.6–1000 μg/mL), and no significant difference was observed compared to the vehicle control.	Coyuco et al. 2011 [37]
Ag-NPs	Size: 20, 40 nm; Surface coating: peptide L-cysteine L-lysine L-lysine	Caco-2	Up to 48 h exposure to Ag-NPs (0–100 μg/ml).	Time-, concentration- and particle size-dependent decrease in cell viability. More toxic effects for 20 nm compared to 40 nm sized Ag-NPs.	Böhmert et al. 2012 [141]
SiO_2_-NPs; ZnO-NPs	Size: 14 and <10 nm for SiO_2_- and ZnO-NPs, respectively; Surface area: 200 and ≥70 m^2^/g for SiO_2_- and ZnO-NPs, respectively; Shape: near-spherical and needle-like ZnO-NPs; Spherical SiO_2_-NPs	Caco-2	Up to 24 h exposure to 0–80 μg/cm^2^ native or digestion simulated (DS) SiO2-, and ZnO-NPs.	SiO_2_-NPs and DS- SiO_2_-NPs reduced cell viability only in undifferentiated Caco-2 cells (even at 5 μg/cm^2^). ZnO-NPs and DS-ZnO-NPs were cytotoxic to both undifferentiated and differentiated cells (24 h)	Gerloff et al. 2013 [45]
TiO_2_-surface treated NPs	T-light SF NPs, a rutile core surrounded by an Al hydroxide layer, vs degradation residues generated after exposure to UV light (T light-DL) or acidic medium (T light-DA). Rutile core size: 7 ± 2 nm × 50 ± 10 nm) Hydrodynamic diameter: T light (347 ± 69); T light-DA (688 ± 209 nm); T light-DL (237 ± 26 nm).	Caco-2	Up to 72 h exposure to TiO2-surface treated NPs (0–100 μg/ml)	No cytotoxic effects were reported using Tripan blue, ATP, XTT and assays	Fisichella et al. 2012 [33]
ZnO- NPs; TiO_2_-NPs	Size: 50–70 nm for ZnO-NPs and <25 nm for anatase TiO_2_-NPs. Purity: 99.7% for TiO_2_-NPs. Mean hydrodynamic diameter in ethanol and serum-free culture medium, respectively: 340.2 ± 12.04, 941.6 ± 118.3 nm for ZnO-NPs; 771.9 ± 110 and 1080 ± 190.5 nm for TiO_2_-NPs. Shape: spherules to rod-like or irregularly shaped particles. Impurities: 0.47% of Cu and traces of Ni, and Pb in ZnO-NPs; 4.0% of Sc, 0.6% of Sb and 0.5% of B in TiO_2_-NPs.	Caco-2	Six and 24 h exposure to ZnO, and TiO_2_-NPs (0–140 μg/ml) with or without inactivated foetal calf serum.	A dose-dependent decrease of cell viability after ZnO-NP exposure. The presence of the foetal calf serum strongly reduced ZnO NP toxic effects. No effect on cell viability was reported after treatment with TiO_2_-NPs either in presence or in absence of foetal calf serum.	De Angelis et al. 2013 [40]
Ag-NPs; TiO_2_-NPs; ZnO-NPs	Size: 20–30, 21, 20 for Ag, TiO_2_, and ZnO-NPs, respectively; Purity: >99.5% for all NPs; Specific surface area: ~20, 50 ± 15, 50 m^2^/g for Ag, TiO_2_, and ZnO-NPs, respectively. Size in Caco-2 media: 202–227, 311–305, and 212–260 nm for Ag, TiO_2_, and ZnO-NPs, respectively. Size in SW480 media: 207–221, 306–300, and 288–303 nm, respectively.	Caco-2 and SW480	Up to 48 h exposure to Ag, TiO_2_, and ZnO-NPs (0–100 μg/ml).	ZnO-NPs (10 and 100 μg/ml) were cytotoxic to both cell lines at 24 and 48 h exposure. No alterations were induced by Ag- and TiO_2_-NPs.	Abbot and Schwab, 2013 [39]
TiO_2_- nanobelts; MW-CNTs	TiO_2_ anatase nanobelts size: length (7 μm), width (0.2 μm), thickness (0.01 μm); Hydrodynamic size (water): 2897 ± 117 nm; Surface area: 17.94 m^2^/g. MW-CNTs size: length (5–10 nm), diameter (20–30 nm). Hydrodynamic size (water): 858 ± 58 nm. Surface area: 513 m^2^/g; Impurities: 1.8% Ni and 0.1% Fe.	Caco-2/HT29-MTX co-culture	One and 24 h exposure to TiO_2_- nanobelts; MW-CNTs (10 and 100 μg/ml)	TiO_2_-nanobelts: only low levels of toxicity were observed. MW-CNTs: no toxicity at 1 h post exposure, and a low level of toxicity (<20% compared to controls) at 24 h post exposure (only for 100 μg/ml).	Tilton et al. 2014 [51]
TiO_2_-NPs	Size: 21 nm (P25 Degussa); 10–25 nm (anatase); 30 nm (rutile); Hydrodynamic diameter (Milliq water): 7.1 ± 4.1, 42.3 ± 14.4 and 88.3 ± 34.1 nm for P25, anatase and rutile, respectively.	Caco-2	Twenty-four h exposure to TiO_2_-NPs (1 μg/ml).	No alterations in cell viability were detected by low LDH leak, and normal cell morphology.	Gitrowski et al. 2014 [53]
TiO_2_-NPs	Size: 12 ± 3 nm anatase NPs (95%); Hydrodynamic diameter (water): 132 ± 0.8 nm.	Caco-2 mono-culture, Caco-2 and HT-29 and Caco-2 and Raji co-cultures.	Forty-eight h exposure to TiO_2_-NPs (0–200 μg/ml).	Exposure to TiO_2_-NPs did not cause overt cytotoxicity. No apoptosis was observed.	Brun et al. 2014 [15]
Ag-NPs, ZnO-NPs	Size: ~90 nm for both NPs.	Caco-2	Twenty-four h exposure to Ag-, and ZnO-NPs (0–200 μg/ml).	Ag- and ZnO-NPs significantly inhibited cell proliferation, with greater effects induced by ZnO-NPs (LD_50_ for ZnO-NPs: 0.431 μg/ml).	Song et al. 2014 [43]
Ag-NPs; Au-NPs	Size: < 100 nm	Caco-2	Twenty-four h exposure to Ag-, and Au-NPs (0–1000 μg/ml).	A dose-dependent toxic effect of Ag-NPs, with IC_50_ values of 16.7 and14.9 μg/ml derived from the MTT and trypan blue exclusion assays, respectively. Au-NPs did not cause any significant decrease in the cell viability.	Aueviriyavit et al. 2014 [47]
Ag-NPs	Mean primary size: 7.02 ± 0.68 nm; Hydrodynamic diameter in acqueous suspension: 14.7 ± 0.2 nm.	Caco-2	Up to 48 h exposure to primary or digested Ag-NPs (0–100 μg/ml).	Digested and undigested Ag-NPs decreased the cell viability of Caco-2 cells in a concentration-dependent manner. No differences emerged between NPs.	Böhmert et al. 2014 [13]
Ag-NPs	Size: 10–100 nm; Size distribution: less than 10% deviation from the primary size; Shape and surface chemistry: spherical NPs stabilized with citrate; Impurities in NP suspensions: none; Agglomeration status: none in culture medium.	LoVo	Up to 48 h exposure to Ag-NPs (0–10 μg/ml).	Cell viability (24 h): At 10 μg/ml, the mitochondrial activity significantly decreased to 53% and to 85% compared to controls for cells exposed to 10 and 20 nm Ag-NPs, respectively. Cell viability (48 h): At 10 μg/ml, 10 nm Ag-NPs mitochondrial activity was reduced to 8% compared to controls. On average, 20–100 nm Ag-NPs resulted in a decrease to 40%.	Miethling-Graff et al. 2014 [105]
Ag-NPs	Size: 20 nm; Hydrodynamic size of Ag-NPs by (A) intensity-weighted distribution (27.3 nm) and (B) by volume-weighted distribution (21.4 nm); Average size by TEM: 20.4 nm.	Caco-2	Three h exposure to Ag-NPs (0–20 μg/ml).	A significant concentration (10–20 μg/ml) -dependent decrease in cell viability compared with controls.	Sahu et al. 2014 [142]
Ag-NPs	Size: < 20 nm	Caco-2 and Raji B cells in co-culture	Twenty-four h exposure to Ag-NPs (0–90 μg/ml) with or without phenolic compounds.	Ag-NPs decreased significantly cellular viability starting from 30 μg/ml with an EC_50_ of ca. 40 μg/ml. Kaempferol (10 or 50 mM) had a protective effect at lower concentrations of Ag-NPs (up to 14%). Resveratrol had no effect.	Martirosyan et al. 2014 [69]
SiO_2_-NPs	Size: 50, 100, 200 nm;	Caco-2	Six h exposure to SiO_2_-NPs incubated in fasting or fed state simulated gastric fluids (0–10 mg/ml).	Up to 6 h time point, no cytotoxicity was observed for all sized NPs. During additional incubation time with fresh medium (24 and 48 h), only 50 nm NPs dispersed in PBS or in fasting simulated fluids, induced a significant cytotoxicity.	Sakai-Kato et al. 2014 [17]
SiO_2_-NPs	Size: 15 and 55 nm; Size distribution range by TEM: 10.8–29.8 and 41–121 for 15 and 55 nm NPs; Shape: spherical.	Caco-2	Twenty-four h exposure to SiO_2_-NPs (0–256 μg/ml).	SiO_2_-NPs (55 nm): a decrease in cell viability (30%) was only observed at the highest tested dose (256 μg/ml). SiO_2_-NPs (15 nm): viability was 80% of controls at 32 μg/ml and 20% at 256 μg/ml. IC50: 43 μg/ml.	Tarantini et al. 2015a [44]
TiO_2_-NPs	Size: 12 ± 3 nm (anatase), 22 ± 4 nm (rutile); Surface area: 82 ± 3 and 73 ± 5 g/m^2^ for anatase and rutile, respectively Hydrodynamic diameter (water): 132 ± 1 nm (anatase); >1000 nm (rutile)	Caco-2	Twenty-four h exposure to TiO_2_-NPs (0–200 μg/ml).	Neither anatase, nor rutile NPs induced overt cell toxicity.	Dorier et al. 2015 [55]
TiO_2_-NPs; SiO_2_-NPs	Size: 22–26 nm ± 10 nm (hydrophilic and hydrophobic TiO_2_-NPs); 14 ± 7, and 13 ± 6 nm for SiO_2_-NPs. Surface areas: 51, 56 (TiO_2_-NPs); 189.2 and 203.9 (SiO_2_-NPs) m^2^/g.	Caco-2	Three and 10 day exposure to TiO_2_-NPs and SiO_2_-NPs (100 μg/ml)	Three day exposure: TiO_2_-NPs did not induce significant changes in the CFE of cells compared to controls. Cytotoxic effects were registered only for 13 ± 6 nm for SiO_2_-NPs (99% SiO_2_), with values of cytotoxicity (CFE = 66% ± 4) Ten day exposure: significant cytotoxic effects were detected after hydrophilic TiO_2_-NPs (CFE = 72% ± 5) and 13 ± 6 nm SiO_2_-NP exposure (CFE = 43% ± 4).	Farcal et al. 2015 [56]
TiO_2_-NPs; ZnO-NPs	Size: 50–70 for ZnO-NPs, and <25 nm for TiO_2_-NPs; Size by TEM: 45–170 (ZnO-NPs) 20–60 nm (TiO_2_-NPs) Hydrodynamic diameter in in cell culture medium without foetal calf serum: 942 ± 118 (ZnO-NPs); 1080 ± 190 nm (TiO_2_-NPs). Purity: 99.7% for anatase TiO_2_-NPs.	Caco-2	Six and 24 h exposure to ZnO, and TiO_2_-NPs (0–128 μg/ml)	Only ZnO-NPs exert a strong cytotoxic effect on cells as determined by replication indexes.	Zijno et al. 2015 [60]
ZnO-NPs	TEM size: 20 to 250/50 to 350 nm; Size in medium: 306 nm; Surface area: 14 m2/g.	Caco-2	Exposure to ZnO-NPs and ZnO-NPs in co-exposure to palmitic acid or free fatty acids	Dose dependent cytotoxic effects were detected for ZnO-NPs (EC50: 25 μg/ml I MTT assay). Co-exposure of ZnO-NPs and palmitic acid to cells showed the largest cytotoxic effects as indicated by the lowest EC_50_ value (19 μg/ml), whereas free fatty acids had a higher EC_50_ value (24 μg/ml).	Cao et al. 2015 [70]
TiO_2_-NPs	Size: 99 ± 30 and 26 ± 12 nm anatase NPs; Hydrodynamic diameter in water: 233 ± 12 and 497 ± 137 nm for the larger and smaller NPs, respectively. Hydrodynamic diameter in culture medium: 719 ± 56 and 727 ± 9 nm for the larger and smaller NPs, respectively. Purity: over 99%.	Caco-2	Twenty-four h exposure to Native NPs and pretreated with digestive fluids (50 and 200 μg/ml)	After 24 h exposure, native NPs do not induce any clear loss of viability on cells. Pretreated NPs are non-toxic to differentiated Caco-2 cells, while can induce a decrease in viability of the undifferentiated Caco-2 cells after 24 h exposure, although the viability remains higher than 86%.	Song et al. 2015 [59]
SW-CNTs; MW-CNTs; MWCNT-OH, and MWCNT-COOH.	Size: SW-CNTs ranged between 1.04–1.71 nm; the layer of MW-CNTs is about 8.4 (± 0.9) graphite layers.	Caco-2	Up to 72 h exposure to CNTs (0–100 μg/ml).	No significant decrease of cell viability was observed at 0.1, 1 and 10 μg/mL doses of four types of CNTs from 4 to 8 h, but the long-lasting treatment (>24 h) increased the cytotoxicity	Chen et al. 2015 [66]
Ag-NPs	Size (untreated NPs): mean radius: 3.2 ± 0.1 nm; width: 1.1 ± 0.3 nm; Size in culture medium (untreated NPs): mean radius: 3.6 ± 0.1 nm; width: 1.2 ± 0.6 nm; Size (digested NPs): 16.0 ± 0.1 nm and 6.6 ± 1.3 nm (with) and 16.6 ± 0.2 and 7.3 ± 1.7 nm (without cell culture medium).	Caco-2	Twenty-four h exposure to untreated or digested Ag-NPs (0–100 μg/ml).	In up to 40 μg/ml Ag no reduction of viability was observed for both NPs. At concentrations higher than 40 μg/ml digested and undigested particles were almost equally cytotoxic.	Lichtenstein et al. 2015 [38]
Ag-NPs	Size: 50 nm; The average size of Ag-NPs by TEM and DLS was 44.7and 54.9 nm, respectively; TEM images demonstrated no noticeable aggregation, agglomeration.	Caco- 2	Four h and 24 h exposure to Ag-NPs (0–50 μg/ml).	A significant concentration (10–50 μg/ml) -dependent decrease in cell viability compared with controls.	Sahu et al. 2016 [143]
Ag-NPs	Size: < 20 nm.	Caco-2 and Raji B cells in co-culture	Three h exposure to Ag-NPs (0–90 μg/ml) with or without a phenolic compound.	Ag-NPs induced a dose-dependent decrease in cell viability. Co-administration with Quercetin protected the cells from the toxic effects of Ag-NPs.	Martirosyan et al. 2016 [68]
Ag-NPs	Size: 10–110 nm.	T84	Fourty-eight h exposure to Ag-NPs (20 and 100 μg/ml).	Little to no change in cell viability compared to controls (acridine orange/ethidium bromide staining). Significant decrease in cell viability only after 100 μg/ml Ag-NPs doses (ATP-based luminescence assay)	Williams et al. 2016 [35]
Ag-NPs	Size: 20 and 200 nm. Hydrodynamic diameter: 129 and 308 for 20 and 200 nm sized Ag-NPs.	Caco-2/TC7:HT29-MTX co-culture	Twenty-four h exposure to Ag-NPs (0–100 μg/ml).	Ag-NPs did not induce cytotoxicity at any of the tested concentrations in single cell lines or in co-culture.	Georgantzopoulou et al. 2016 [22]
PVP capped Ag-NPs; TiO_2_-NPs; Phosphine capped Au-NPs.	Ag-, TiO_2_-, and Au-NP size: < 20; 7–10 and 15, 80 nm, respectively. Mean hydrodynamic diameter in DMEM culture medium: 120 ± 4, 896 ± 133 and 51 ± 6, 116 ± 5 nm, respectively.	Caco-2 mono-, and co-culture with THP-1, MUTZ-3 cells in a 3D model of intestinal mucosa	Twenty-four h exposure to NPs (0–625 μg/cm^2^) in both inflamed and not-inflamed conditions	Au-NPs and TiO_2_-NPs did not affect cell viability. Ag-NPs: the highest concentration induced a significant cytotoxicity in Caco-2 mono-culture > than in co-culture, with no influence due to the inflammatory status.	Susewind et al. 2016 [48]
TiO_2_-NPs	TiO_2_-NP size; 50 and 100 nm (anatase); 50 nm (rutile); 21 nm (P25Degussa); Hydrodynamic diameter (DMEM): 227.78 ± 3.62 (anatase 50); 253.40 ± 4.11 (anatase 100); 194.20 ± 2.14 (rutile 50); 193.85 ± 1.86 nm (P25 Degussa).	Caco-2 cells	Twenty-four, and 72 h exposure to NPs (0–50 μg/ml)	No change in Caco-2 cell viability was evident at 24 h-exposure. 72 h exposure: 50 nm anatase (10, 25, 50 μg/ml), 100 nm anatase (50 μg/ml), 50 nm rutile (50 μg/ml), and P25 Degussa TiO_2_-NPs (25, 50 μg/ml) reduced cell viability.	Tada-Oikawa et al. 2016 [52]
TiO_2_-NPs	Size: < 25 nm anatase NPs (99%); Surface area: 45–55 m^2^/g; Hydrodynamic diameter (water): 604 ± 24 nm.	HT-29	Up to 48 h exposure to TiO_2_-NPs (0–36 μg/ml).	No significant cytotoxic effect of TiO_2_-NPs was observed in LDH and MTT assays at all concentrations after 6, 24, and 48 h exposure.	Ammendolia et al. 2017 [50]

*Caco-2 cells* human colorectal adenocarcinoma cells, *CFE* colony forming efficiency, *COOH- SW-CNTs* carboxylic acid functionalized single walled carbon nanotubes, *CTAB* cetyltrimethylammonium bromide, *DLS* dynamic light scattering, *DS* digestion simulated, *EC*_*50*_ half maximal effective concentration, *HT-29* human colon carcinoma cells, *HT29-MTX* human adenocarcinoma mucus secreting cells, *IC* inhibition concentration, *LD50* Lethal dose, *LoVo* human colon carcinoma cell line, *MUTZ-3* human dendritic cells, *MW-CNTs* multi-walled carbon nanotubes, *PAA* polyacrylic acid, *PAH* polyelectrolyte poly(allylamine) hydrochloride, *PEG* poly-ethylene glycol, *PEG-SW-CNTs* poly(ethylene glycol) functionalized single walled carbon nanotubes, *Raji B line* human Burkitt’s lymphoma cells, *RKO* human colon adenocarcinoma cells, *SW- CNTs* single walled- carbon nanotubes, *SW480* human colon adenocarcinoma cells, *T84* human colonic epithelial cells, *TEM* transmission electron microscopy, *THP-1* Human macrophages

Overall, in vitro data are seemingly discordant, however, as discussed above, different experimental conditions (doses, exposure duration, cell types, functionalization) may at least in part explain the different results. An important potential causal factor is represented by the effective dose cells are challenged with, which may be quite different in experiments using the same nominal dose: ENM administration under static conditions to cells cultured at the bottom of a culture plate may lead to different interaction rate of the materials with the medium and therefore with cells in different experiments, leading to different cellular concentrations. For example, a fraction of ENM may aggregate in liquid suspension and come in contact with cells at relatively fast rate, whereas those suspended may remain in suspension for the whole duration of the experiment and never get in contact with the cellular surface. Even small changes in the proportion between aggregated and suspended ENM may lead to quite different dose-response curves. The ultimate fate of ENM in a fluid is then dictated by its mass density, i.e. nanomaterials will settle if their mass density is greater than that of the fluid [71]. Suggestions to overcome these limitations have recently been discussed [72].

#### In vivo studies

Only a few studies have investigated toxicity of ENM in vivo. Studies focusing on *Ag ENM* provided evidence for liver inflammatory infiltration after acute and chronic administration [73–76], although studies demonstrating no toxic effects have also been reported [77–79]. However, the difference is mainly related to the dose used. Indeed, in one of the studies demonstrating ENM adverse effects on the GIT [74], a NOAEL (no observable adverse effect level) of 30 mg/kg and LOAEL (lowest observable adverse effect level) of 125 mg/kg were calculated. In studies showing no toxicity, doses lower than the calculated LOAEL were used.

Interestingly, in positive studies, the liver damage was elicited at comparatively lower doses in mice than in rats.

*TiO*_*2*_ *ENM* were found to induce inflammatory changes in the small bowel [80] and also to enter the systemic circulation to accumulate and cause inflammation and oxidative damage in the liver, kidney and spleen [81–86]. However, other studies did not detect any adverse effect after oral administration of titanium dioxide, even at very high doses [87–89].

In order to reconcile the contradictory data regarding TiO_2_ ENM, Warheit and Donner [88] noted that negative studies had been performed according to OECD test guidelines, whereas those showing adverse effects were “experimental-type” studies, and highlighted the predominant use of mice in studies indicating adverse effects and of rats in those showing no effects, suggesting that differences in susceptibility of exposed animals may contribute to the final result. In addition, commercial test materials were used in studies showing no effects, whereas “home-made” particles were more often used in studies in which adverse effects were observed. However, the presence of substantial adverse effects at doses as low as 1 mg/kg/bw reported by Tassinari et al. [86], and their absence at doses three order of magnitude higher reported by Warheit et al. [88] remains hard to be explained.

Local intestinal damage was reported after oral ingestion of *Carbon nanotubes* (*CNTs*). Indeed, multiple necrotic foci in the small intestine were observed after a 30-days treatment with multi-walled carbon nanotubes (MWCNTs) in mice, maybe related to the direct CNT-mediated mechanical damage to the enterocytes [90]; whereas a 6 month chronic exposure to MWCNTs in rats induced a dose-dependent decrease in the number of villi in the small intestine characterized by apical necrosis [91]. Ingested *ZnO ENM* were reported to undergo size-dependent intestinal absorption with accumulation in multiple organs and damage to liver and pancreas [92–95]. Finally, ingested *SiO*_*2*_ *ENM* caused low-level hepatotoxicity in rats following a 10-week exposure [96]. As highlighted above, no observed adverse effect levels (NOAEL), which might be extrapolated to exposed workers, were calculated for some of the studies following OECD guidelines [73, 87–89, 92]: for silver ENM a NOAEL of 30 mg/kg per day was extrapolated [73], whereas for ZnO the calculated NOAEL was 268 mg/kg. NOAEL ranging from 1000 mg/kg to 24,000 mg/kg have been proposed for titanium dioxide [88].

### Mechanisms of toxicity

Mechanisms of ENM induced toxicity have been recently reviewed [20, 97] and will not be reported in detail here. We will, however, discuss two developing new fields represented by the interaction of ENM with the gut microbiota and by the contribution that the “omics” technique may give to detect effects which are not observed by using traditional approaches. In the second section of this review, we will also discuss the possible different toxicity mechanisms occurring after direct ingestion of ENM or indirect ingestion, following ENM inhalation.

#### Effects on intestinal microbiota

Most of the functions of the gastrointestinal tract are facilitated, influenced or modulated by the vast resident collection of microbes, known collectively as the intestinal microbiota [98]. The intestinal microbiome has been a major topic of research in the fields of microbiology and medicine [46, 99, 100] and only recently it has been considered in the context of potential toxicological effects of ingested metals, including their nanoforms [64, 78]. Given that a disruption of the normal intestinal microbiota, also known as dysbiosis, has been linked to severe medical conditions like colitis, inflammatory bowel disease, diabetes and metabolic syndrome, determining whether ENM have an impact on commensal gut microbiota is an essential step in evaluating their overall safety [101].

Few data are available from human. For instance, Das et al. [102] found that the human microbiota (evaluated in stool samples) could be significantly impacted in metabolic activity, as demonstrated by the reduced total gas produced by the stool microbial ecosystem as well as in phylogenetic assemblages, since the anaerobe, Gram negative abundance was significantly reduced by a subacute 48 h exposure to 25–200 μg/ml Ag ENM.

Studies in rodents evaluated the effects of ingested Ag ENM on the gut microbiota, although with non-univocal results [78, 100]. Williams et al. [64] reported a significant decrease in colony-forming units of indigenous ileal microbial populations of rats sub-chronically gavaged with 10–110 nm PVP-coated Ag ENM at doses of 9, 18 and 36 mg/kg bw/day for 13 weeks. The most pronounced effects on cultivable bacteria were observed at lower doses and with smaller diameter particles. Importantly, when real-time PCR was utilized to amplify DNA extracts, i.e. 16 s universal bacterial gene, to measure the relative expression of bacteria, no significant differences could be detected in any of the treatment groups. This may be due to the fact that 16 s–based real-time PCR technique, although proposed as the most suitable method for the quantification of specific microbial communities compared to the traditional culture strategy or the next generation sequencing, is not able to distinguish live bacteria from uncultivable dead or non-proliferating microbes. Therefore, caution should be paid in the interpretation of such kind of data. They also compared the ratio of Bacteroidetes to Firmicutes, the two major phyla of the intestinal microbiome, showing that 110 nm Ag ENM at the highest dose induced a significant increased ratio due to a decrease in Firmicutes. However, no clear description was available concerning the physiologic effect, either detrimental or beneficial, of these alterations in animals.

Another in vivo study [103] performed in mice, showed that Ag ENM could affect the gut microbiota at doses relevant for human dietary exposure (0.046–4.6 mg/kg). In fact, a 28 day oral exposure to Ag ENM mixed in food increased the ratio between Firmicutes and Bacteroidetes phyla inducing a dose-dependent decrease in Bacteroides and an increase in Firmicutes as assessed by the next generation sequencing technique [103]. The trend in Firmicutes alterations reported in this study [103] was different compared to that emerged in Williams et al. [64], maybe in relation to the different techniques employed to analyse the microbiota. Interestingly, when 4 or 8 month aged Ag ENM were used to treat animals, microbiome alterations could not be confirmed. These ENM, in fact, induced a less evident, if any, inversion of the Firmicutes to Bacteroidetes ratio. Ag ENM sulfidation, as a major transformation process for ENM in contact with organic materials, was demonstrated to be responsible for the reduction of aged Ag NP ENM solubilization and Ag + ion release, that may all prevent the gut microbiota alterations observed with freshly prepared Ag ENM.

A polydisperse mixture of 60–100 nm Ag ENM (0–100 μg Ag/kg for 4 h) incubated with ileal contents sampled from weaned piglets, induced a dose-dependent reduction in intestinal coliforms [104]. However, in the same study, when pigs were treated with 20–40 mg Ag/Kg for 2 weeks, only a non-significant trend toward coliform reduction could be detected.

These results were in contrast with those obtained by Hadrup et al. [78] in Wistar rats and Wilding et al. [100] who found in C57BL/6NCrl mice that 28 days gavage administration of 14–110 nm Ag ENM (2.25–10 mg/kg bw/day) irrespective of their coatings, i.e. PVP or silver acetate, did not affect the balance and number of the two major bacterial phyla in the gut [78].

Interspecies differences in intestinal pH, gut microbiota, diet as well as pathological conditions, which may affect microbial composition generating significant inter-individual variation, even in genetically identical animals with identical starting microbial populations, may explain such different outcomes. Certainly the ENM physico-chemical diversity, in terms of size, coating, or other physicochemical properties may have a different antimicrobial activity [7]. Additionally, the chemical transformations undergone by ENM in aging consumer products as well as during digestion processes may all affect the potential risk for microbial alterations in real human conditions of exposure, particularly in relation to the ENM solubilization ability. In this perspective, to assess the degree, rate and duration of ion release over time, also in in vitro models, should be verified as an interesting instrument to predict the fresh or aged ENM potential to affect microbial communities. Finally, the experimental methodologies utilized in microbiota investigations should be considered as a possible confounding issue for the direct comparison of the data [103].

Sample type, collection site, the employment of a culture strategy or not, lab techniques for the microbiota analysis based on totally different approaches may all affect the final outcomes of the studies and should be carefully considered for an adequate interpretation of the results.

#### Effects detected by “omic” techniques

To gain insights into potential mechanisms of action of ENM exposure on intestinal cells, biochemical changes have been investigated by using “omics-” aproaches. By using this technique, transcriptional effects involving an enrichment of gene ontology categories related to unfolded proteins, chaperons and stress responses were detected after 5 μg/cm^2^ ZnO ENM exposure for 4 h of Caco2 cells [41]. As far as epithelium permeability is concerned, Brun et al. [15] demonstrated a significant up-regulation in the expression of genes encoding proteins involved in the maintenance of cell junctions in Caco-2 and Caco-2-HT29-MTX models exposed to 50 μg/mL of TiO2 ENM for 6 h or 48 h; similar findings, showing up-regulation of several genes involved into tight junction and desmosome formation were reported after exposure of T84 cells to Ag ENM (100 μg/mL for 48 h) [35]; by contrast a significant down-regulation of genes encoding junctional proteins was observed by Brun et al. [15] in the ileum of mice exposed to a single gavage of 12.5 mg/kg TiO_2_ ENM, and sacrificed 6 h after the gavage. These seemingly conflicting results may at least in part be related to the different times of exposure, which may allow, in the case of relatively protracted exposure, the induction of compensative mechanisms of repair.

In terms of nanosafety implications, genes whose expression levels change significantly in a manner that correlates with the effects of the ENM-exposure might be useful as early nanotoxicity biomarkers.

As far as the mechanisms involved in the oxidative stress are concerned, it has been reported the concomitant down regulation of mammalian mitochondrial proteins, and the up-regulation of those involved into the cellular redox systems after exposure of LoVo cells to 10 μg/ml for 24 h Ag ENM [105]. On the other hand, up-regulation of cytosolic proteins associated with anti-oxidant activities has been found, this finding being probably related to the development of compensatory mechanisms [106].

Promising results have been obtained when using the omics technique in order to discriminate between the effects related to metallic ENM and those due to the release of ions: as an example, a higher number of deregulated proteins was detected after exposure to Ag NPs compared to the ionic form [107].

Whether or not distinct pathways may be activated in response to specific ENM has been investigated by Tilton et al. [51], who performed global transcriptome and proteome analyses of intestinal (Caco-2/HT29-MTX) co-cultured cells, exposed to 10 and 100 μg/ml TiO_2_ nanobelts (TiO2-NBs) and multi-walled carbon nanotubes (MWCNT). Interestingly, the early 1 h post-exposure transcriptional response was primarily independent of ENM type, showing similar expression patterns in response to both TiO_2_-NB and MWCNTs, while the 24 h response was unique to each nanomaterial type. TiO_2_-NB treatment affected several pathways, such as those associated with inflammation, apoptosis, cell cycle arrest, DNA replication stress and genomic instability, while MWCNTs regulated pathways involved in cell proliferation, DNA repair and anti-apoptosis.

Finally, the “omics” technique has also been exploited in order to identify the mechanisms underlying the different responses sometimes elicited by ENM of different size. It has been recently reported that 20 nm sized Ag ENM (1 μg/ml for 24 h) regulated different sets of proteins, principally involved in pathogen-like response and in the maintenance of the intestinal barrier function and integrity, with a distinct pattern of cellular responses compared to 200 nm Ag particles at the same experimental conditions in a co-culture of Caco-2/HT29-MTX cells [22].

## Impact of the inhaled enm on the git and occupational implications

### GIT is a relevant target for extrapulmonary effects of inhaled ENM

**Fig. 1:**
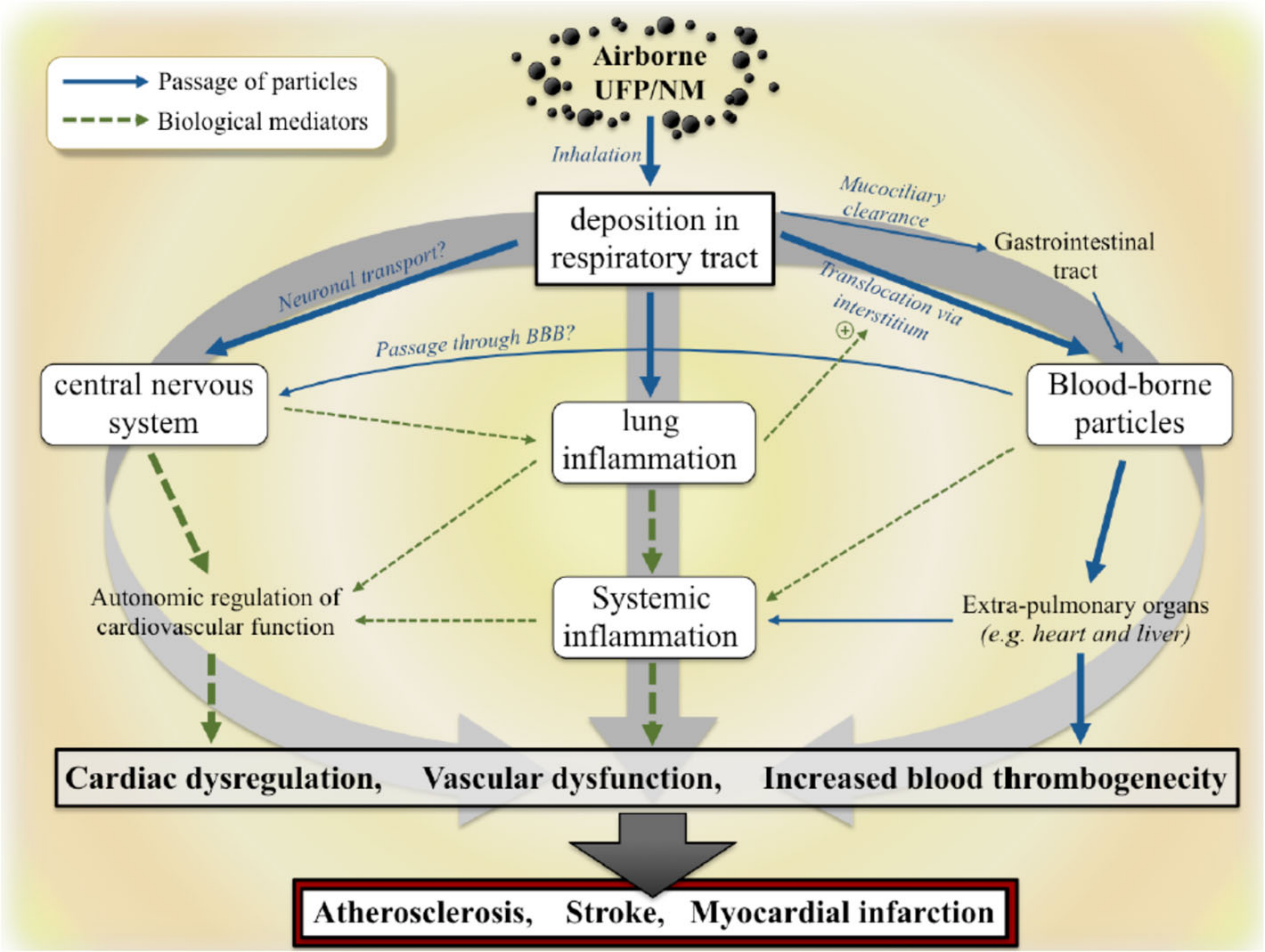
Mechanisms of extrapulmonary effects of inhaled ENM. Legend: UFP = Ultrafine particles; NM = Nanomaterials. Reproduced from Environmental Health Perspective [140] (https://ehp.niehs.nih.gov/EHP424/)

Inhalation is the main route through which people, in particular workers, may come in contact with ENM, and the lung is therefore the most obvious target of their possible toxic effects. However, in recent years, a lot of extrapulmonary effects of inhaled ENM, regarding almost all organs and organ systems, have been reported [108]. As summarized in Fig. 1, these effects may be related to direct mechanisms (i.e. due to nanoparticles crossing the alveolo-capillary barrier) or to indirect mechanisms (i.e. due to the release of toxic mediators following nanoparticles/lung interaction). It is important to note that translocation to the systemic circulation is very low, below 0.5% of the exposure concentration [109], however, in the case of chronic exposure, accumulation of nanoparticles in target organs might reach a critical threshold causing injury.

### GIT: An important overlooked target of extrapulmonary effects of inhaled ENM

Among extrapulmonary effects, those on the gastrointestinal tract have not explored yet. This is surprising, because inhaled nanoparticles may reach the gastro-intestinal tract at a much larger amount than other organs. In fact, like other organs and organ systems, the GI tract can be exposed to nanoparticles crossing the alveolar barrier and reaching the systemic circulation, as suggested by the substantial fecal excretion of intravenously-injected ENM [93]. The amount of ENM reaching the gut through the systemic circulation is probably greater than that reaching other sites, as shown by Lee et al. who found that silver ENM were transferred from systemic circulation into the gut at a much higher rate than into the kidney or other biological sites [110]. In addition to ENM crossing the aveolar-capillary barrier (the only mechanism of direct effect for other organs), the GIT may be also exposed a) to inhaled ENM cleared from the lung through the muco-ciliary escalator (which is a major clearance pathway for ENM from the lung as compared with translocation through the alveolo-capillary barrier [111] and b) to nanoparticles directly ingested while breathing air (the so called “aerophagia”). People affected by this common disorder ingest air (and its content) at a much higher rate than normal persons [112].

The relevance of gastro-intestinal exposure following ENM inhalation is strongly supported by the recent finding that after pulmonary exposure of rats to CeO2 ENM, the highest amount of ENM was recovered from feces (71–90%), ENM recovered from the lungs being 7–18%, whereas urine and other extra-pulmonary organs both contributed between 4 and 6% of the total recovered mass [113]. Of note, the presence of ENM in feces is by itself the proof of a significant interaction with the GIT, since it implies a contact with the intestinal microbiome/microbiota, a major player in GI physiology and pathology [114, 115].

As reported for other organs and organ systems, there is evidence that the gut may be sensitive to mediators released by the inflamed lung, the so-called lung-gut axis. This may be the case for interleukin-6 (IL-6), which is systemically elevated in patients with emphysema [116] and is implicated in the pathogenesis of inflammatory bowel disease [117, 118]. In patients with asthma, histopathological and functional alterations of the gastro-intestinal tract have been described [119], probably related to the circulation of activated lymphocytes between the mucosal tissues of the lungs and of the gastrointestinal tract [120].

As shown in Fig. 1, systemic inflammation has been reported after pulmonary exposure to ENM [121], and it is considered a major pathophysiological mechanism in order to explain the extrapulmonary effects of ENM. On the basis of the above reported evidence, these effects are to be expected also for the GI tract after inhalation of nanoparticles.

In summary, on the basis of the currently available evidence, not only the direct and the indirect mechanisms evoked for the effects on other extrapulmonary sites are plausible for the gastro-intestinal tract, but their impact might be even greater for this biological site in comparison to others.

### Peculiar effects on GIT of inhaled ENM in comparison to ingested ENM

The reasons why the possible effects of inhaled ENM on the GI tract have been neglected until now are probably two: from one side the lack of substantial epidemiological evidence of relevant gastrointestinal effects in workers inhaling particles of larger size; from the other one, the fact that the very large amounts of nanoparticles ingested with food and drinks seem not to cause substantial damage.

As far as the first argument is concerned, only sparse data linking exposure to particulate and functional [122] or organic [123] GI diseases are available. Indeed, a systematic investigation of this possible association has not been performed. In any case, it should be considered that ENM may have enhanced or novel toxic properties in comparison to the same material in the bulk form, therefore the lack of robust epidemiological data for the inhaled bulk form cannot be translated to inhaled nanoparticles.

The assumption that ingested ENM are not harmful (second point), is questioned by recent experiments showing that ingested ENM may cause important consequences on the homeostasis of the GI tract, in particular on the gut microbiome, starting a chain of events leading to significant physiological and anatomical alterations [7].

In addition, it should be considered that the biocorona of inhaled nanoparticles is quite different in comparison to that of ingested nanoparticles: the first are primarily covered by biomolecules of the fluid lining the respiratory tract, whereas the biocorona of the second ones is mainly determined by the proteins, lipids and carbohydrates present in the food, which they are usually ingested with. The different biological identity between inhaled and ingested nanoparticles may be associated with quite different biological effects, given the increasing awareness of the role of biocorona in governing the activity of nanoparticles in living organisms [124].

As an example, in experiments exploring the biological fate of nanoparticles ingested with food, it was found that gold nanoparticles ingested with milk are decorated with beta lactoglobulin, a protein of bovine milk, and that the protein is totally displaced by bile salts in the small intestine (whose excretion from the gallbladder into the intestine is in turn stimulated by food ingestion) so that a complex formed by a core of gold nanoparticles and a surface of bile salts is formed [125]. This complex resembles the complex lipid droplet/bile salts, which allows the absorption of lipids through the intestinal epithelium, otherwise not permeable to them. It can therefore be speculated that a similar phenomenon may occur for inorganic nanoparticles ingested with milk, allowing their transport through the intestinal epithelial cells.

On the other hand, the bio-corona of inhaled nanoparticles is characterized by a relatively fixed pattern of phospholipids derived from the contact with the pulmonary surfactant, whereas the protein composition changes according to the surface properties of the inhaled particles [126]. Nothing is known about the interaction of this nanoparticle/biocorona complex with the biological fluids of the gastrointestinal tract, (which are in any case of different composition than those encountered by nanoparticles ingested with food, due to the lack of food-related stimulation of biliary and pancreatic secretions) and we suggest that this topic should be explored (see recommendations).

As highlighted above, pristine nanoparticles (i.e. nanoparticles without a pre-formed biocorona) can also be ingested with aerophagia. These nanoparticles are covered in the stomach with a protein corona mainly composed by pepsin, a proteolytic protein secreted by the gastric chief cells. This protein seems to influence the aggregation status of silver nanoparticles, which may have implications on their toxicity [127]. Furthermore, the presence of a pepsin corona might explain the reported lack of antimicrobial effect of silver nanoparticles in the distal murine intestine [100].

A third type of biocorona may characterize ENM reaching the GIT through the blood after pulmonary pulmonary exposure: in this case ENM are covered with a biocorona primarily formed in the lung and subsequently modified in the blood [128]. Table 2 summarizes the different biocorona composition of nanoparticles reaching the gastro-intestinal tract through different modalities, and highlights possible biological effects.

**Table 2:** Different bio-corona and biological effects of nanoparticles reaching the gastro-intestinal tract through different modalities

Modality	Primary bio-corona (before coming in contact with the GI tract)	Available information on changes of biocorona during GI Transit	Effects on biokinetics/activity	Relative amount of nanoparticles
Ingested with food	Derived from interaction with food	Biocorona formed with the milk protein beta-lactoglobulin may be replaced by biliary salts in small intestine	The complex nano-particle/biliary salts may be efficiently absorbed in the small intestine.	High
Ingested with air (aerophagia)	No primary biocorona	A pepsin biocorona is formed in the stomach.	May affect the agglomeration status of nanoparticles	Low
Ingested after muco-ciliary clearance	Formed with surfactant phospholipids and proteins in the lung.	Unknown	Unknown	Substantial
Reaching the GI tract through systemic circulation (after lung crossing)	Formed with surfactant phospholipids and proteins in the lung. It may be partly modified by the contact with blood proteins (128) and by the contact with bile before being excreted in the gut	Unknown	Unknown	Low

Another important point to be taken into consideration is that nanoparticles reaching the gut following inhalation may have a synthetic identity different from that of ingested nanoparticles. As an example, some nanoparticles at high risk of being inhaled (e.g. carbon nanotubes) have a low chance to reach the gastrointestinal tract through ingestion. Therefore, not only the same nanoparticle may have different effects on the GI tract, depending on the modality of exposure, but also some nanoparticles reaching the gut following inhalation may have a low chance to do so by ingestion with food and may therefore cause biological responses which cannot be observed with ingested nanoparticles.

## Discussion

The increasing interest in nanomaterials for advanced technologies, consumer products, and biomedical applications has led to great excitement about potential benefits, but also concerns over the potential for adverse human health effects. The gastrointestinal tract represents a likely route of entry for many nanomaterials. In occupational settings, gastrointestinal exposure may result from the mucociliary clearance of inhaled nanomaterials, or from a direct exposure in case of accidental events or when proper standards of personal and industrial hygiene are not met [129].

The gastrointestinal epithelium and supporting elements primarily act as a physical and biochemical barrier between the luminal compartment and the interior of the human body [130]. A key factor important to understand the gastrointestinal toxicological profile of ENM is the complex “interplay” between the great variability in ENM physico-chemical properties and the absolutely changeable conditions found along the gastrointestinal system. ENM chemical composition, structure/cristallinity, size and size distribution, shape, concentration, surface area, functionality and charge may all vary according to the methods of ENM production, preparation processes, and storage, but may also be modified when ENM are introduced into biological compartments. A number of gastrointestinal luminal parameters, such as physical forces, osmotic concentration, pH, digestive enzymes, (i.e. buccal amylase, gastric pepsin, and intestinal pancrease and lipase), together with different gastrointestinal transit time, dietary status, other biochemicals and commensal microbes may potentially impact ENM properties therefore affecting their toxicological profile. In this scenario, future researches should provide a systematic and deeper characterization of both the primary physico-chemical features of ENM and those secondarily acquired through the interactions occurring along the gastrointestinal tract, e.g. the degree of aggregation or agglomeration and the percentage of available ions for those ENM undergoing dissolution, known influencing factors of NP toxicity. Moreover, investigations focused on the possible toxic impact of ENM on the gastrointestinal system should elucidate which parameters are the strongest inducers of any changes in ENM features, and, on the other side, whether the full range of nanomaterials may be modified in the gastrointestinal milieu, or if only certain categories of ENM are subjected to such modifications [97]. Due to their intrinsically increased surface/mass ratio, ENM may adsorb biomolecules on their surface upon contact with food and/or biological fluids in gastrointestinal compartments, resulting in the formation of a biomolecular “corona” which may affect the uptake, bioaccumulation and biotransformation of NPs possibly leading to unanticipated, reduced or augmented, toxicities [131, 132]. All these aspects should be carefully considered to better correlate ENM primary and acquired properties and biological effects, in order to support the production of “ENM safe by design” that, while maintaining most of the innovative and revolutionary ENM features may, at the same time, be characterized by lower toxicity [133].

Additionally, M cell- targeting of ENMs should be carefully considered as another possible pathway of interaction between ENMs and the intestinal milieu which may have possible systemic implications. M cells are specialized epithelial cells of the gut-associated lymphoid tissues (GALT) that can play an immunosensing and surveillance role by delivering luminal antigens through the follicle-associated epithelium to the underlying immune cells. Recent evidence has supported the critical function of endogenous and synthetic nanomineral chaperones in the efficient transport of molecules across the epithelial barrier of the lymphoid follicles in the small intestine [27, 31]. In this perspective, further investigation should be focused to assess whether ENMs may be involved in protecting molecules from the GI degradation, favoring an effective M-cell delivery, and a greater transfection efficacy, therefore promoting tolerogenic or stimulatory immunological responses. Overall, this may be important to define the role of ENMs in vaccine delivery systems for priming more effective humoral and mucosal immune responses in the hosts [134].

A challenging issue relates also on the difficulties to extrapolate experimental data to realistic human/occupational exposure contexts. In vitro studies demonstrated the ability of several types of ENM to induce cytotoxic, inflammatory, oxidative stress as well as genotoxic responses in exposed cells. However, in vitro models may not accurately resemble the complexity of the in vivo response [135]. Therefore, in the attempt to improve physiological relevance of in vitro models and better mimic in vivo gastrointestinal situations, including conditions of inflamed mucosa, multi-cellular cultures have been proposed. These may incorporate mucus secreting goblet cells [34], microfold-cells [61], and even immune-competent macrophages and dendritic cells [136] and have shown a diverse, as well as more predictive of in vivo response, susceptibility to the ENM injuries compared to the cellular monolayers [48]. Moreover, for the assessment of the toxicity of orally ingested ENM, additional refinements, for instance, pre-treatment or co-administration of particles with gastrointestinal reconstituted bio-fluids or food matrix components, may be employed in order to achieve more meaningful in vitro tests, with the aim to deeply understand how protein corona changes may affect ENM uptake, metabolism and toxicological behavior.

In vivo studies, on the other hand, can provide information concerning ENM toxico-kinetics in gastrointestinal and extra-intestinal tissues and ENM toxico-dynamic behaviors in relation to their physicochemical properties. In this regard, future in vivo studies should overcome the difficulty to extrapolate findings from the generally, higher-doses, short-term investigations on animal models, to real low-dose, long-term conditions of exposure experienced in general living and occupational settings, through the adoption of more realistic experimental designs. Moreover, in vivo studies should provide useful data to identify possible biomarkers of exposure and early effect as well as indicators of susceptibility to greater ENM induced adverse health outcomes. Macrophage-mediated mucociliary escalation followed by fecal excretion is a pathway for clearing the inhaled NPs from the body [129, 137]. Although it is rather difficult to routinely employ feces as a suitable biological matrix for occupational biomonitoring, on the account of the aforementioned clearance mechanism, in the case of metal- or metal oxide-NP exposure, the measurement of the elemental metal content in feces should be viewed as a means to evaluate the recent/current exposure to this kind of NPs [129]. Moreover, future investigations should explore possible biomarkers of early effect, particularly as concerns mucosal inflammatory alterations, which may be detected in fecal matrix. Clinical experience, carried out with inflammatory bowel diseases, Chron’s disease or ulcerative colitis, in this sense, may provide useful suggestions for potential biomarkers to be investigated and validated in the nano-toxicological gastrointestinal field [138]. Additionally, taking advantage of more innovative “omics techniques”, a comprehensive analysis of differential gene and protein expression should be performed to derive molecular profiles indicative of NP exposure or early effect which may also explain possible early modes of cellular response to NPs. This may be helpful to understand also biological processes affected by ENM or possibly involved in their toxico-dynamic behavior to identify potential parameters of individual susceptibility to ENM adverse effects [139]. Importantly, in the attempt to define conditions of greater susceptibility to ENM adverse outcomes, intra- and inter-individual differences in normal physiology as well as in specific diseases should be deeply analyzed. These conditions, in fact, may alter the gastrointestinal environments affecting ENM stability and movement as well as epithelial permeability. Age, gender-specific differences, pregnancy status, malnutrition, sleep cycle and stress, as well as inflammatory bowel diseases can all result in increased lining permeability and can augment the susceptibility to the absorption of some types of ENM and to the induction of possible toxic effects.

An emerging aspect that deserves deep attention regards the potential interactions of ingested ENM with the gut microbiota [7].

Few studies are still available concerning such interesting issue, and some of them showed conflicting results. In this field, some knowledge gaps should be overcome by future investigations, particularly concerning which pathological consequences may derive from microbiota alterations induced by ENM. In an opposite perspective, alterations in ENM toxico-kinetic and dynamic profile caused by the same microbiota as well as by pre-existing altered microbial states, such as gram negative bacterial overgrowth, should be clarified. To deeply assess such issues, fecal samples as representative models of the microbiota of the colon, together with samples of the human small intestine microbiota obtained from ileostomies of people undergoing colon surgery, may be used. Moreover, the employment of «humanized» models by the inoculation of human gut microbiota to gnotobiotic animals should be carefully considered as an ideal model to study in vivo effects of ENM in order to transfer animal data to humans. The study of the interactions between ENM and the gastrointestinal tract may provide the identification of innovative biomarkers based on the possible specific modifications induced by ENM on the gut microbiota. However, confounding effects related to individual characteristics, pathological statuses, diet, drugs and co-exposure to other xenobiotics should be taken into careful consideration to adequately interpret these results.

Overall, this information would provide deep insight into possible ENM toxicological aspects that have not been sufficiently explored up to date, with the aim to reach a suitable assessment of risks in general living and occupational ENM exposure settings.

In this perspective, another crucial aspect which needs to be adequately explored in the future is represented by the possible gastro-intestinal effects and gastrointestinal-mediated systemic effects of inhaled ENM. There is evidence that GIT may be a relevant target for extra-pulmonary effects of inhaled ENM, and that these effects may be different (and possibly more relevant) than those induced by ingested ENM. In this respect, experimental studies focused to this specific topic are needed. We recommend in particular:

1. Assessing the impact of GI fluids and (gut) microbiome on the biocorona of particles that are deposited in the respiratory tract and after mucociliary clearance being swallowed versus nanoparticles ingested with food and how this affects the biodistribution
2. Assessing the toxic effects of inhaled nanoparticles (i.e. incubated with pulmonary surfactant) on gastric cells, cells of the small intestine and cells of the colon (including the interaction with the microbiome), as compared with toxic effects of nanoparticles ingested with food using in vitro methods
3. Perform a systematic comparison of effects of inhaled nanoparticles on the gastrointestinal tract and on intestinal microbiome compared with ingested nanoparticles.

The results of these studies might be the basis for refining the focus on possible effects of ENM on human at high risk of lung exposure (i.e. workers directly or indirectly involved in nanotechnology).

## Conclusions

The gastro-intestinal tract (GIT) is considered to be a potential target of ENM ingested with food and water. It is believed that the possible biological effects on the gastrointestinal tract (GIT) deriving from ENM ingestion involve mainly the consumers, whereas workers may be only marginally affected, the inhalation being the main way through which they may come in contact with ENM. The biological effects of ENM on this organ are poorly known both because of inherent difficulties in their assessment due to the complex GIT environment and because most available experimental studies suggest the lack of overt toxicity.

In this review we discussed the most relevant gaps in the knowledge of the biological effects of ENM on the GIT and demonstrate that, by logically connecting the available sparse information on this topic, it is possible to identify sequential key processes, spanning from the alterations of intestinal permeability to functional and organic cellular damage, which may shed light on the pathophysiological mechanisms of the gut/ENM interaction.

We also re-interpreted the results of some experiments, such as, for example, the presence in the stools of almost the total amount of ingested ENM, a finding generally considered to be an indicator of the lack of substantial local and systemic effects of the ingested ENM; however, the recent evidence that ENM may have a relevant impact on the gut microbiota, even in the absence of substantial contact with GIT cells, indicates that local and systemic biological effects mediated by changes in gut microbiota are possible even in this situation.

Last but not least, we challenged the common belief that the possible biological effects of ENM on the GIT are confined to consumers, showing that inhaled ENM, which represent the main route of ENM exposure for workers, may induce peculiar and substantial effects on the GIT: these effects may be different (and potentially more important) than those related to ingested ENM.

Taken together, our findings suggest that the GIT should have a primary role in the future research on the biological effects of ENM. In this light, we identified and suggested proper experimental protocols aimed to verify this hypothesis.
